# Perceptual salience influences food choices independently of health and taste preferences

**DOI:** 10.1186/s41235-019-0203-2

**Published:** 2020-01-03

**Authors:** Jane Dai, Jeremy Cone, Jeff Moher

**Affiliations:** 1 Department of Psychology, Williams College, 18 Hoxsey St., Williamstown, 01267 MA USA; 2 Department of Psychology, Connecticut College, 270 Mohegan Avenue, New London, CT 06320 USA

**Keywords:** Visual salience, Taste preference, Health, Attention

## Abstract

**Background:**

Making decisions about food is a critical part of everyday life and a principal concern for a number of public health issues. Yet, the mechanisms involved in how people decide what to eat are not yet fully understood. Here, we examined the role of visual attention in healthy eating intentions and choices. We conducted two-alternative forced choice tests of competing food stimuli that paired healthy and unhealthy foods that varied in taste preference. We manipulated their perceptual salience such that, in some cases, one food item was more perceptually salient than the other. In addition, we manipulated the cognitive load and time pressure to test the generalizability of the salience effect.

**Results:**

Manipulating salience had a powerful effect on choice in all situations; even when an unhealthy but tastier food was presented as an alternative, healthy food options were selected more often when they were perceptually salient. Moreover, in a second experiment, food choices on one trial impacted food choices on subsequent trials; when a participant chose the healthy option, they were more likely to choose a healthy option again on the next trial. Furthermore, robust effects of salience on food choice were observed across situations of high cognitive load and time pressure.

**Conclusions:**

These results have implications both for understanding the mechanisms of food-related decision-making and for implementing interventions that might make it easier for people to make healthy eating choices.

## Significance

One of the leading causes of preventable death in the United States is obesity. Diet is a well-known risk factor for elevated body mass index, and individual food choices are highly consequential for providing adequate nutrition. Although there is a wealth of scientific knowledge regarding the nutrition, physiology, and biochemistry of food consumption, the direct application of these insights is limited to the clinical treatment of the chronic diseases that arise as complications of obesity. A valuable approach to addressing prevention at the population level is to combine information from psychology and public health. Mechanisms of individual behavioral change can be used to develop practical interventions that promote health. Since health systems all over the world are fighting obesity and diet-related chronic disease, it is increasingly critical for research to identify minimal and cost-effective interventions to promote healthy eating.

Through this research, we examined the role of visual attention on healthy eating intentions and choices. Do people tend to make food decisions based on healthiness or tastiness, and do these behavioral trends change if the scenario is simulated in increasingly real-world conditions? Our results for the robustness of the salience effect are promising and suggest that visual attention is an emerging area for developing interventions that promote healthy eating. Just as research on menu-labelling has shown that this health policy is effective in significantly reducing calorie consumption in large chain restaurants, we anticipate that the work presented in this paper will ultimately inform policy changes that support healthy food decisions.

## Perceptual salience influences food choices independently of health and taste preferences

Obesity and other nutrition-related diseases, such as diabetes, cardiovascular disease, and stroke, are leading contributors to the burden of disease in the United States (Wang, McPherson, Marsh, Gortmaker, & Brown, 2011), contributing $114 billion to the total cost of healthcare (Tsai, Williamson, & Glick, 2011). Diet has been shown to play a substantial role in preventing obesity and its related set of chronic diseases, with nutrition emerging as a crucial determinant of health (Nishida, Uauy, Kumanyika, & Shetty, 2004). The decisions that determine dietary choices are influenced by multiple individual and environmental factors (Kearns, Schmidt, & Glantz, 2016; Story, Kaphingst, Robinson-O'Brien, & Glanz, 2008).

To examine eating choices in the laboratory, we drew on previously established cognitive paradigms that model real-world choice behavior for preference decision-making. Typically, these studies present two choices and ask participants to choose their preferred option (e.g., Krajbich, Armel, & Rangel, 2010). One theoretical framework for these binary choice tests is the binary-attribute attentional drift diffusion model (baDDM), which uses fixation patterns between pairs of relevant stimuli to predict purchasing decisions (Fisher, 2017). An important feature of baDDM is that it assumes that choices between competing alternatives for consumption are made by accumulating evidence in favor of each alternative, and that once evidence in favor of one of the choices exceeds a critical threshold, it is chosen and the other is rejected. Importantly, the extent to which evidence in favor of a choice alternative accumulates is based, in part, on the order of acquisition. In the context of food decisions, choices are biased by where individuals look, such that people are more likely to choose items that are attended to for longer and have stronger associations (Roininen, Arvola, & Lähteenmäki, 2006).

In one study, this approach was applied to food decisions by having participants choose their preferred food from a pair of appetitive junk food images (Armel, Beaumel, & Rangel, 2008). Each picture appeared in alternating order, with one being presented for a shorter duration than the other for a total of six times to imitate the natural phenomenon of alternating eye fixation that occurs when an individual chooses between two competing items. Participants were more likely to choose foods that were displayed for longer, suggesting that longer exposure—and therefore, more time spent attending—to a food item can bias decisions towards that item. However, the same did not hold for pairs of aversive food items, suggesting that longer exposure may not be relevant when decisions are made between unappealing choices.

Visual attention can be influenced in other ways besides alternating presentation. Perceptual salience—the degree to which exogenous features (e.g., visual brightness) contrast with their surroundings—often plays a key role in biasing attention when multiple items are present (e.g., Itti & Koch, 2001; Itti, Koch, & Niebur, 1998; Maljkovic & Nakayama, 1994). In some cases, attention can even be captured by salient items automatically, regardless of an observer’s intentions (e.g., Theeuwes, 1992), and this applies to eye movements as well (e.g., Theeuwes, Kramer, Hahn, & Irwin, 1998), though this is not necessarily automatic and other factors such as current top-down goals (e.g., Bacon & Egeth, 1994; Folk, Remington, & Johnston, 1992) or semantic information within visual scenes (e.g., Henderson, Hayes, Rehrig, & Ferreira, 2018) can override perceptual salience as a driver of attentional focus. Thus, the eyes are likely to spend more time on parts of a display that are perceptually salient (e.g., Parkhurst, Law, & Niebur, 2002). These eye movements may help filter relevant information to inform decision-making (Knudsen, 2007). Since many real-life food decisions are made after visual inspection (e.g., images of menu items and displays in grocery store aisles), our focus on visual attention has direct application to everyday behavior.

To examine the relevance of perceptual salience on food decisions, Milosavljevic, Navalpakkam, Koch, and Rangel (2012) presented observers with two food items and asked them to choose their preferred one. They manipulated the perceptual salience of one of the choices by increasing its brightness relative to both the other choice as well as surrounding non-relevant items. Their findings showed that salience was a predictor of food choices, and at short time exposures, it was an even stronger predictor of food choices than preference. This provides evidence that perceptual salience can influence preference decision-making.

These and other studies (e.g., Enax, Krajbich, & Weber, 2016; Itti & Koch, 2001) have largely manipulated the physical properties of food while keeping the type of food relatively consistent. However, another critical factor in food decisions that has not been studied extensively is the healthiness of a food choice, particularly since both individual consumers and government policies have paid more attention to nutrition in food decisions in recent years (Guthrie, Mancino, & Lin, 2015; Miller & Cassady, 2012; Ollberding, Wolf, & Contento, 2010). Furthermore, in real-world decisions, people must choose between options that vary along multiple dimensions, such as taste and healthiness. Here, too, research has shown that decisions can be manipulated. For example, participants were more likely to choose healthy food options when they were explicitly asked to consider the healthiness of the food immediately preceding a choice trial (Hare, Malmaud, & Rangel, 2011). In recent studies, these two factors have been more directly compared. For example, healthiness information appears to influence decisions more slowly than tastiness information (Sullivan, Hutcherson, Harris, & Rangel, 2015). However, to our knowledge, no studies have simultaneously manipulated both taste preference and healthiness in evaluating the impact of perceptual salience on food decisions. This is an important methodological shift, as the robustness of the salience effect to simultaneous manipulations of factors, such as taste and health, is currently unknown.

Here, we ask whether perceptual salience can bias people towards healthy food decisions even when a healthy option is presented alongside an unhealthy option that is rated as tastier. This question is important for understanding how perceptual salience affects multi-dimensional preference decision-making. From a more practical perspective, this approach may also be informative regarding the extent to which simple visual manipulations might affect real-world food decisions.

We asked participants to rate a series of both healthy and unhealthy food items on taste preference. Next, we presented them with a series of two-alternative forced choice trials involving one healthy item and one unhealthy item. Participants were asked to indicate which food they would prefer to eat. In each trial, either the healthy or unhealthy item had been previously rated by that participant as slightly higher than the competing alternative on taste preference. In other words, each trial pitted two choices against each other, such that one item was rated as slightly tastier than the other. In some trials, either the healthy or unhealthy item was made perceptually salient by increasing the item’s brightness relative to its competing item and its surroundings. In the first experiment, we tested this approach in both an online and an in-person sample. In the second experiment, we tested this approach in multiple contexts that mimic the conditions under which real-world decisions about food are made. In one condition, participants had to make food decisions while simultaneously adding to or subtracting from a number in memory, reflecting a high cognitive load (e.g., Sweller, 1988). In a second condition, a time limit was enforced to examine the effects of salience under more rapid preference decision-making. We also collected data regarding participants’ hunger levels to determine whether hunger influences perceptual salience effects. Because response times can be affected by perceptual salience (e.g., Yantis & Egeth, 1999), we examined response time across all experiments in addition to choice outcomes.

Previous work has shown that in repeated decision-making, an observer’s choice during one decision can inform their choices on subsequent decisions (Sharot, Velasquez, & Dolan, 2010). This can have tangible application in real health interventions, as studies have shown that active cognitive appraisal in a food-cue response task can help with inhibitory control training and reduce the real-world consumption of undesired foods (e.g., Boswell, Sun, Suzuki, & Kober, 2018; Lawrence et al., 2015; Meule et al., 2014). Thus, in the present study, we also examined the effect of prior choices on current choices. These factors may have implications for characterizing the determinants of decisions and habit formation.

By studying the combined effects of perceived health and visual salience, we aim to determine whether salience manipulations can consistently change food choice patterns.

## Experiment 1: Visual saliency bias in competing food decisions

### Experiment 1.1

#### Method

##### Participants

Across all experiments, participants received a monetary payment of approximately $10/h in exchange for completing the study. Participants were screened to ensure that they had no uncorrected vision problems and no color blindness. The protocol was approved by the Williams College institutional review board.

For Experiment 1.1., 50 participants were recruited through Amazon Mechanical Turk (MTurk).^1^ This and subsequent sample sizes were based on similar sample sizes in research examining the impact of salience on food decisions (e.g., Armel et al., 2008; Hare et al., 2011; Krajbich & Rangel, 2011). Participants whose ratings for healthy and unhealthy options were too different, such that we could not create all necessary trial types, were replaced.^2^ The use of MTurk for experimental behavioral research has been validated as a source of data that is comparable to in-person laboratory studies (Crump, McDonnell, & Gureckis, 2013).

##### Stimuli

Participants rated and made decisions on 32 different food items, 16 of which were commonly understood to be junk foods (e.g., candy bars and packaged desserts) and 16 of which were understood to be healthy foods (e.g., bananas and oranges). Images of the selected food items were taken from a database of food images created for experimental research into eating and appetite (Blechert, Meule, Busch, & Ohla, 2014) and presented as high-resolution color images (92 ppi). During the first phase of the experiment, when participants rated the different food items, all stimuli were centrally presented.

During the second phase of the experiment, when participants made decisions between competing food items, each trial began with a fixation cross in the center of the screen measuring 20 pixels by 20 pixels. The two food images—one unhealthy item and one healthy item—were placed symmetrically on opposite sides of the display. Each item was surrounded by eight gray circles as placeholders, both to simulate a real food choice from a crowded display and to facilitate the manipulation of salience. This design was largely adapted from Milosavljevic et al. (2012). Each image was placed 200 pixels away from the central fixation cross, and each image measured 320 × 240 pixels. The gray circular placeholders had a radius of 20 pixels and were offset by 90 pixels from the center of the food image along the *x*-axis and 70 pixels along the *y*-axis. Whether the healthy or unhealthy item was on the right was randomized for each trial. On 50% of the trials, which were selected randomly, one of the items was made perceptually salient by increasing the image brightness from 30% to 100% of the maximum brightness using the brightness settings in JavaScript.^3^ In trials where both images were non-salient, the brightness of both images was 30%. The gray circles were always presented at 30% brightness. Thus, the increase in brightness increased the contrast between the food item and the surrounding gray circles, making the food item more perceptually salient.

All programming for this design on MTurk was done using custom programs through psiTurk (Gureckis et al., 2016).

##### Procedure

Following a design adapted from Hare et al. (2011), participants were first asked to indicate their perceived taste preferences for all 32 food stimuli by rating each item on a scale of 1 (not very tasty) to 5 (very tasty). The prompt “Please rate each food on a scale from 1–5, where 1 is the least tasty and 5 is the most tasty” appeared at the beginning of this section, followed by the food images, with alternating healthy and unhealthy items. Ratings were used to measure subjective values of perceived taste preference.

**Fig. 1:**
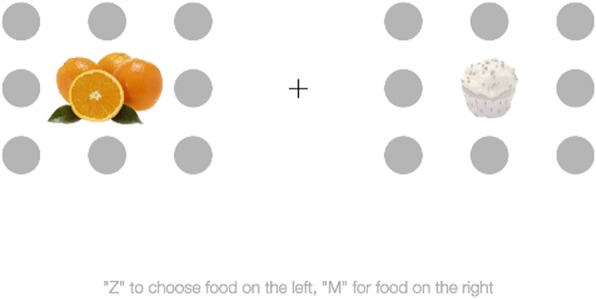
Sample image of a two-alternative forced choice test. In this trial, the healthy food stimulus is salient

Participants then spent 10 to 15 min completing a set of 200 two-alternative forced choice tests between pairs of food stimuli (Fig. 1). The design of the comparison tests followed that in a study on visual saliency bias by Milosavljevic et al. (2012). Each trial began by displaying a central fixation cross for 1000 ms, which stayed on the screen while the food stimuli appeared.

The food choice pairing in each choice trial was defined by each participant’s subjective ratings of junk and health food items. One healthy and one unhealthy food image were paired together for each choice trial. To ensure that each trial involved a difficult food decision, the difference between the taste ratings of the two foods was one scale point. For example, a trial might include an apple that was rated as a 4 and a cupcake that was rated as a 5. Half of the trials set the healthy food as the better tasting option, and the other half set the unhealthy food as the better tasting option. In addition, half of the trials included one salient object. In these trials, the salient object could be the healthy or unhealthy option. The salience and taste rating conditions for each trial were randomly ordered throughout the experiment.

Food stimuli were presented on the screen for an unlimited amount of time, although participants were instructed to make decisions as quickly as possible, as previous studies have demonstrated that salience manipulations are most robust under quick response conditions (e.g., Milosavljevic et al., 2012). Participants chose which food they would most like to eat by pressing the “Z” key to choose the food item on the left or the “M” key to choose the food item on the right.^4^

##### Data analysis

Across all experiments, our primary dependent variable was selection rate, which reflected how frequently participants in each condition chose the healthy option. To examine the overall selection rate, we conducted a 3 × 2 repeated-measures analysis of variance (ANOVA) with factors of salience type (nothing salient [NS], healthy salient [HS], or unhealthy salient [US]) and taste preference, as defined by whether the healthy item received a higher or lower taste score than the competing unhealthy item (healthy tastier [HT] vs. unhealthy tastier [UT]). We also conducted a 3 × 2 × 2 ANOVA on selection rate with salience type, taste preference, and an additional factor of previous decision to examine sequential effects (healthy-healthy, unhealthy-healthy, healthy-unhealthy, and unhealthy-unhealthy) and whether a participant’s food choice on a prior trial influenced their food choice on the current trial. Note that this analysis cannot support causal infererences, as we did not randomly assign each participant to make a particular choice on any given trial. Still, this may be informative regarding whether recent behavior is a useful predictor of current behavior for food-related decisions. Some participants made either all healthy (or all unhealthy) decisions in a particular condition. These participants were not included in analyses of sequential patterns of behavior in any experiments. We report only main effects and interactions involving previous decision for these analyses, as other components of the ANOVA are redundant with earlier analyses. Finally, we conducted a 3 × 2 × 2 ANOVA with factors of salience type, taste preference, and decision type (healthy vs. unhealthy) on response times to determine whether the speed of the decision, rather than the choice itself, was also impacted by these factors. Participants who did not make any responses in any one of the 12 experimental conditions were not included in this analysis for any of the experiments. Where the assumption of sphericity was violated, we used Greenhouse–Geisser corrected *p* values for all analyses across all experiments.

#### Results

##### Selection rate

Not surprisingly, the selection rate of the healthier of the two food stimuli was higher when the healthy item was rated tastier (.71, HT) than when the unhealthy food was rated tastier (.29, UT), *F*(1, 49) = 102.02, *p* < .001, *η*_*p*_^*2*^ = .68.

**Fig. 2:**
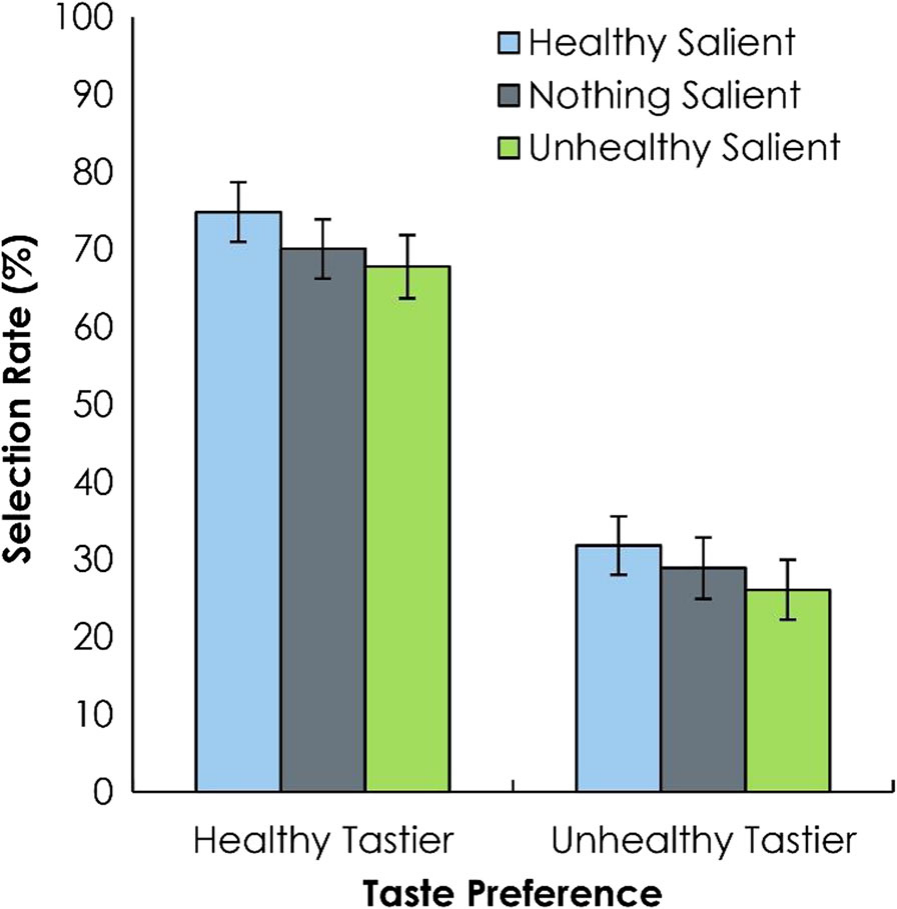
Experiment 1.1. Visual saliency bias in two-alternative forced choice tests on MTurk. Selection of the healthy stimulus varied by main effects of salience type and taste rating. Error bars reflect the standard error of the mean

In addition, there was a significant effect of salience type, *F*(2, 98) = 16.82, *p* < .001, *η*_*p*_^*2*^ = .26 (Fig. 2). We conducted post hoc least significance difference (LSD) contrasts to evaluate the comparisons among the three levels of salience: NS vs. HS, NS vs. US, and HS vs. US. The selection rate (probability of choosing the healthy item) was higher in the HS condition (.53, HS) compared to the NS condition (.50, NS), *p* < .001. Selection rate was also higher in the HS condition compared to the US condition (.47, US), *p* < .001. Selection rate was also higher in the NS condition compared to the US condition, *p* = .012. Finally, there was no interaction between salience type and taste preference, *F*(2, 98) = 0.56, *p =* .574, *η*_*p*_^*2*^ = .01.

Together, these results demonstrate that visual salience affects food decisions even when both the health and taste preferences of the presented foods are varied. The lack of interaction suggests that the impact of salience is independent of these other factors, which may otherwise have influenced food decisions. In other words, regardless of taste preference and health considerations, food decisions are biased towards items that are salient and thus, likely to capture attention.

##### Sequential effects

Research has shown that decisions can be influenced by recent prior choices (e.g., Hermans, Spruyt, De Houwer, & Eelen, 2003; Tipper, 1985). This holds important implications for the mechanisms behind habit formation, particularly if this type of sequence effect can be demonstrated based on just a single previous decision. In other words, are people more likely to choose a healthy option if they had chosen a healthy option in the preceding trial? We found no main effect of previous decision, *F*(1, 43) = 2.70, *p =* .107, *η*_*p*_^*2*^ = .06 and no interactions were observed, *F*s < 2.17, *ps* > .127. This suggests that each decision was treated largely independent of previous decisions.

##### Response time

We were also interested in examining the extent to which choice response time was impacted by both salience and taste rating. We conducted a 3 × 2 × 2 repeated-measures ANOVA with factors of salience type (NS, HS, and US), taste rating (HT and UT), and decision type (healthy and unhealthy) on response time. There were no main effects or interactions, *Fs* < 2.71, *ps* > .112. This may have been an artifact of the lack of response time pressure, which would naturally increase the variance in the response time measure, as there can be both wide overall inter-individual variation and significant intra-individual variation in response times.

### Experiment 1.2

The results of Experiment 1 suggest that salience plays a potent role in food decisions, independently of other factors such as healthiness and taste preference. However, an important limitation of these findings is that they were based on purely hypothetical decisions. That is, at no point was the participant presented with a choice that would actually result in them receiving the food they had chosen. To address this limitation, in the next experiment, we used the same paradigm in a lab study in which the decisions that participants made had actual consequences. We informed participants that they would actually receive one of the food items they chose during the experiment.

#### Method

##### Participants

During this phase of the study, 31 participants were recruited from the undergraduate population at Williams College (*M*_age_ = 20.2 years, standard deviation [SD] = 1.4 years, range 18–23 years; 20 female, 11 male). All participants received $5 USD as compensation.

##### Stimuli and procedure

The procedure was nearly identical to Experiment 1.1. However, participants were informed that they would actually receive their chosen food item from one of the two-alternative forced choice tests at the end of the experiment. Specifically, one of the trials paired an orange and a Kit Kat chocolate bar regardless of how the participant had rated the two foods during the taste-rating task, and their choice on this trial determined whether they received one or the other at the end of the experiment. (Data from this prearranged trial were not included in analyses.) Participants were not told which trial would actually be executed and did not know that it was predetermined. Because participants did not know which trial would be executed in advance, they presumably should have treated all decisions as potentially having real consequences. Similar procedures have been used in other studies and have been shown to be incentive-compatible (e.g., Sullivan et al., 2015).

#### Results

##### Selection rate

**Fig. 3:**
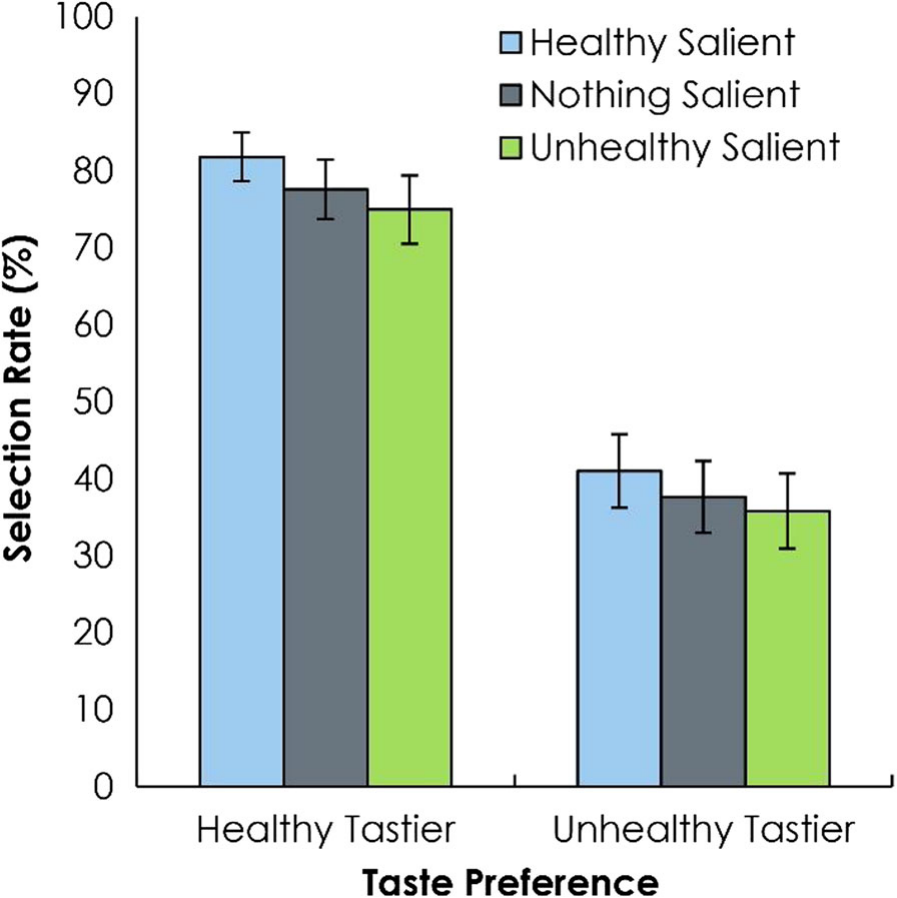
Experiment 1.2. Visual saliency bias in two-alternative forced choice tests with one reinforced choice. Selection of the healthy stimulus varied by salience type and taste rating. Error bars reflect the standard error of the mean

As in Experiment 1.1, the selection rate of the healthier of the two food stimuli was higher when it was rated tastier (.78, HT) than when the unhealthier food was rated tastier (.38, UT), *F*(1, 30) = 97.88, *p* < .001, *η*_*p*_^*2*^ = .77. There was also once again a main effect of salience type, *F*(2, 60) = 7.12, *p* = .002, *η*_*p*_^*2*^ = .19 (Fig. 3).

We again conducted post hoc LSD contrasts to evaluate the differences among the three levels of salience type. The selection rate was higher in the HS condition (.61) compared to the US condition (.55), *p* = .003. It was also higher compared to the NS condition (.58), *p* = .010. Selection rate in the NS condition was not significantly higher than in the US condition, *p* = .151. However, the qualitative pattern closely matched that of Experiment 1.1. We conducted an additional ANOVA with data from both experiments with experiment included as a between-subjects factor. This showed no interactions between either factor or experiment, *ps* > .747. Thus, the results appear to be quite similar across both the lab-based and online-based experiments, providing evidence that online participants treat the decisions as they would if they knew the decisions might be enforced.

As in Experiment 1.1, no interaction was found, *F*(2, 60) = 0.19, *p =* .787, *η*_*p*_^*2*^ < .01.

##### Sequential effects

Again, there was no main effect of previous decision, *F*(1, 28) = 2. 83, *p =* .104, *η*_*p*_^*2*^ = .09, and no interactions were observed, *Fs* < 1.96, *ps* > .161.

##### Response time

As in Experiment 1.1, a 3×2×2 repeated-measures ANOVA revealed no main effects, *Fs* < .69, *ps* > 0.415. We observed an interaction between taste preference and decision type, *F*(1, 21) = 21.00, *p* = .004, *η*_*p*_^*2*^ = .33. No other interactions were observed, *Fs* < 1.88, *ps* > 0.178.

### Discussion

The results of Experiment 1 demonstrate that food decisions between two competing food items, one healthy and the other unhealthy, are influenced by perceptual salience. When the healthier food was made visually brighter and thus salient, participants selected it more frequently, even if it had been rated less tasty than the competing option. Whereas previous work has suggested that salience can influence target selection in decisions, these results demonstrate that salience is a potent factor in food-related decisions and that it operates independently in the presence of multiple other factors that typically influence choice, such as taste and healthiness. Although these effects may appear small, resulting in an approximately four percent increase in the likelihood of selecting the more salient alternative, they are nonetheless likely to be practically significant. For example, a four percent reduction in caloric intake could result in approximately 560 fewer calories per week, which would translate to approximately 10 lb of fat over a year. Thus, a small, cost-free intervention could have significant consequences for public health.

Another key finding is that we observed consistent results between both the online study involving hypothetical decisions and the lab study involving real decisions with real consequences, suggesting that the choices of participants in online samples do not meaningfully differ from those made by undergraduates in the lab. Thus, for the remaining study, we rely on an MTurk sample.

## Experiment 2

In Experiment 1, we found robust effects of salience even when other factors such as tastiness and healthiness were manipulated. Our main goal in Experiment 2 was to assess this salience effect in contexts that more closely resemble the way that such decisions are made in real-world choices. First, actual food decisions frequently occur under conditions of high cognitive load. For example, a shopper in a grocery store may also be thinking about returning home so that they can drive their child to soccer practice and what they have to do at work tomorrow. Research has shown that cognitive load can reduce performance on a simultaneous task and reduce the ability to exert cognitive control over a decision (Speier, Valacich, & Vessey, 1999). Furthermore, a number of studies have shown that a high cognitive load can increase the degree to which attention is influenced by perceptual salience (e.g., Lavie, Hirst, de Fockert, & Viding, 2004). Thus, in one condition, we manipulated cognitive load to test whether increased cognitive load affects the influence of salience on food decisions.

Another factor relevant for real-world food decision-making is that food decisions are often made quickly because people are in a hurry. Previous research has shown that for rapid decision speeds, salience bias influences decisions more than preferences (Milosavljevic et al., 2012), and that time limits in general can directly affect choice behavior (e.g., Reutskaja, Nagel, Camerer, & Rangel, 2011; Suter & Hertwig, 2011). Thus, in another condition, we introduced a time limit to assess its impact on the magnitude of the salience effect in food-related decision-making.

The broader goal of the second experiment was to test the generalizability of the salience effect found in the first experiment. That is, does cognitive load or time pressure impact the salience effect? Alternatively, is the salience effect robust across multiple contexts?

The experimental design, sample size, exclusion criteria, and analysis plan for this study were pre-registered prior to collecting data. This information along with all data reported in the present manuscript are available from the Open Science Framework (OSF) at: https://osf.io/8q259.^5^

### Method

#### Participants

In total, 279 participants were recruited on MTurk. Of these, 53 did not complete the experiment because they quit early, had technical problems, or rated foods such that the experiment could not create the necessary conditions for the second phase. The final sample was *N* = 226 (*M*_age_ = 36.2 years, SD = 10.5 years, range 18–70 years; 128 female, 97 male, 1 declined to respond).

#### Procedure

We randomly assigned participants to one of three conditions in a 3 (conditions: control, cognitive load, and reaction-time deadline) × 3 (salience types: NS, HS, and US) × 2 (taste preferences: HT and UT) design. The procedure for the control condition was identical to Experiment 1.

The procedure in the cognitive load condition was identical to the control condition, except that we additionally manipulated cognitive load using a modification of a procedure used in prior work (Milosavljevic et al., 2012). The color of the central fixation cross was manipulated as part of the cognitive load task. Participants were told to begin with the number 100 in active memory. Before each trial, the central fixation cross was colored either blue or red. If the cross was blue, participants added 1 to their current score, whereas if the cross was red, they subtracted 1. As in the previous experiments, participants were asked to make a food decision at the end of each two-alternative forced choice tests. However, they were required to keep track of the score and report it at the end of the experiment. Each participant saw 120 increments and 80 decrements (chosen ahead of time so that the correct answer was not an easy guess, such as 100).

At intervals of 40 trials, participants were asked “What is your current score?” and received immediate feedback on the accuracy of their answers. Participants had an average error of *M* = 7.2 (SD = 10.2) on these responses, suggesting that they were not randomly guessing but rather making an effort, albeit imperfectly, to maintain the correct score.

In the reaction-time (RT) deadline condition, the procedure was also identical to the control condition, with one exception: we introduced a time limit on all food choices of 1.5 s. This value was chosen to induce pressure to respond while still giving participants time to consider their options. Previous research has used brief displays of this length or shorter in similar food-choice tasks (e.g., Milosavljevic et al., 2012), so we chose this limit to create meaningful time pressure. If participants did not respond within that time, the stimuli disappeared and the data from that trial were discarded.

We conducted the same analyses as Experiment 1, with experimental condition (control, cognitive load, and RT deadline) as an additional between-subject factor. At the conclusion of the experiment, we also asked participants to rate their hunger level on a scale from 1 to 7. These data were intended for exploratory analyses and are not discussed further in the present manuscript but are available from the OSF website listed above.

### Results

#### Selection rate

As in Experiment 1, the selection rate (probability of choosing the healthy item) was higher when the healthy item was rated tastier (.65, HT) than when the unhealthy item was rated tastier (.38, UT), *F*(1, 223) = 214.63, *p* < .001, *η*_*p*_^*2*^ = .49.

**Fig. 4:**
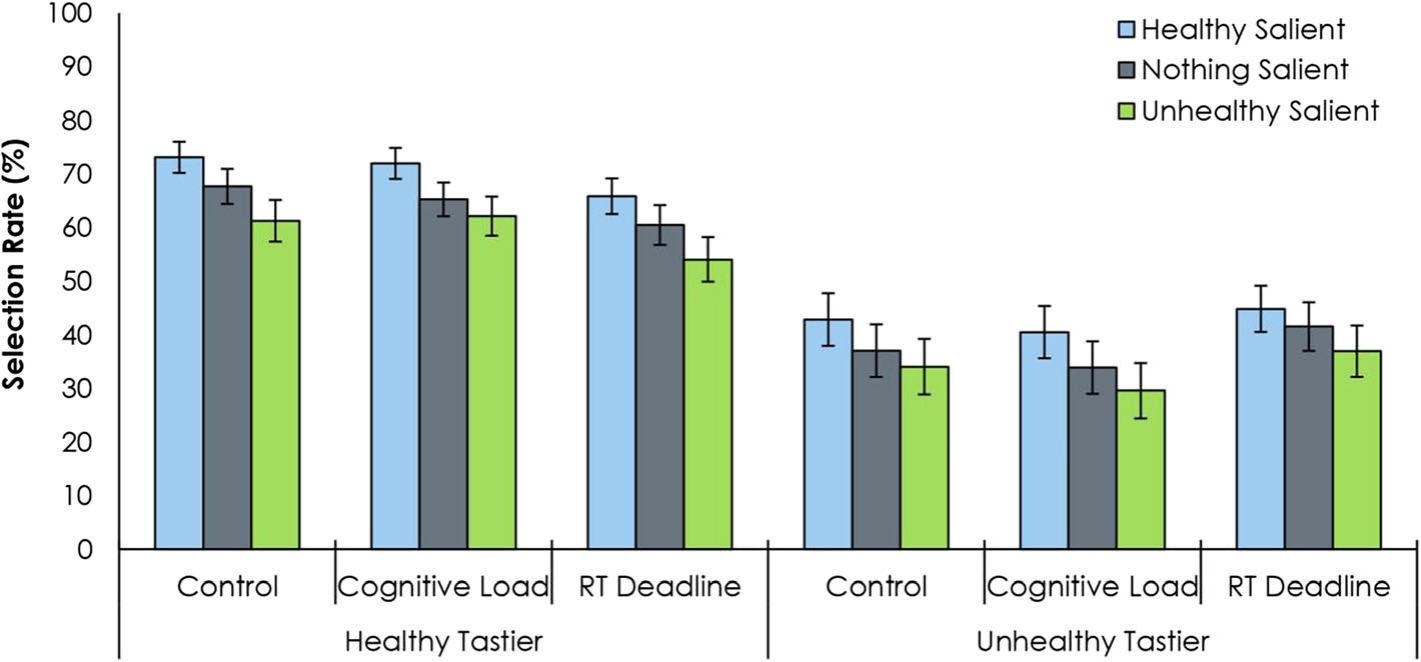
Visual saliency bias in two-alternative forced choice tests with one reinforced choice. Selection of the healthy stimulus varied by salience type and taste rating. We also varied the conditions under which participants made their decisions by inducing cognitive load or enforcing a reaction time deadline. Error bars reflect the standard error of the mean. RT reaction time

There was once again a main effect of salience type, *F*(2, 446) = 89.32, *p* < .001, *η*_*p*_^*2*^ = .29 (Fig. 4). Post hoc LSD contrasts found that all comparisons were significant, *ps* < .001. The selection rate of the healthier of the two food stimuli was higher when the healthy food was salient (.57, HS) compared to when no item was salient (.51, NS), which in turn was higher than when the unhealthy item was salient (.46, US). Again, there was no interaction between taste preference and salience type, *F*(2, 446) = 1.80, *p =* .173, *η*_*p*_^*2*^ < .01.

Next, we analyzed the effects of experimental condition (simple comparison, cognitive load, or time pressure). There was no main effect of experimental condition on the selection rate, *F*(2, 223) = 0.19, *p =* .829, *η*_*p*_^*2*^ < .01, and no interaction between experimental condition and salience, *F*(4, 446) = 0.43, *p =* .788, *η*_*p*_^*2*^ < .01. There was an interaction between taste preference and experimental condition, *F*(2, 223) = 4.66, *p* = .010, *η*_*p*_^*2*^ = .04. This was primarily driven by a smaller bias towards the higher rated choice in the RT deadline condition (.60, HT; .41, UT) compared to the control condition (.67, HT; .38, UT) and the cognitive load condition (.67, HT; .35, UT). There was no three-way interaction, *F*(4, 446) = 1.41, *p =* .237, *η*_*p*_^*2*^ = .01.

#### Sequence effects

Unlike Experiments 1.1 and 1.2, we found a main effect of previous decision, *F*(1, 200) = 8.60, *p* = .004, *η*_*p*_^*2*^ = .04. The selection rate of the healthier of the two food stimuli was higher when participants had previously made the healthy decision (.49) compared to when participants had previously made the unhealthy decision (.47). No interactions were significant, *Fs* < 1.47, *ps* > .227.

The significant effect of previous decision here suggests the possibility that one decision about which food to choose may predict, to some extent, the next decision that a person will make. However, because these are not manipulated independent variables, we cannot draw any conclusions about the causal direction of this relationship. While we did not find a significant effect of previous decision in Experiments 1.1 and 1.2, it is notable that the effect size in those experiments was higher in magnitude compared to Experiment 2, and the magnitude of the difference between the previous healthy choice and previous unhealthy choice conditions was also numerically larger in those experiments compared to Experiment 2. Thus, the lack of statistical significance in the first experiment is likely attributable to a lack of statistical power due to a smaller sample size. Still, we should note that the effect size of this sequence effect is relatively modest, particularly in comparison to the robust salience effect that was observed across all experiments.

#### Response time

We found a main effect of salience type on response times, *F*(2, 316) = 3.86, *p* = .027, *η*_*p*_^*2*^ = .02. Post hoc LSD contrasts showed that response times were faster when the healthy item was salient (1098 ms) compared to the unhealthy item being salient (1338 ms), *p* < .036, or no items being salient (1382 ms) conditions, *p* = .002. Response times were not significantly different when the unhealthy item was salient compared to no items being salient, *p* = .721.

There was also a main effect of taste rating, *F*(1,158) = 7.37, *p* = .007, *η*_*p*_^*2*^ = .05. Response times were faster when the healthy item was the higher rated item (1160 ms) compared to when the unhealthy item was the higher rated item (1386 ms). Together, these results both suggest that choices are made more rapidly when contextual factors, such as previous expressions of preference or physical salience, favor the healthy choice.

Finally, as expected, there was a main effect of condition, *F*(2,158) = 20.52, *p* < .001, *η*_*p*_^*2*^ = .21, with the shortest response times in the RT deadline condition (695 ms). The control condition was slower (1499 ms), and the cognitive load condition was slowest (1624 ms). Post hoc LSD contrasts revealed that response times in the RT deadline condition were shorter than the other two conditions, *ps* < .001, but that the control condition and cognitive load condition did not differ, *p* = .463. No other main effects or interactions were significant, *Fs* < 1.55, *ps* > .187.

### Discussion

The results of Experiment 2 provide additional evidence that food decisions between two competing healthy and unhealthy food items are robustly affected by perceptual salience. Even when other factors are experimentally manipulated, such as cognitive load or RT deadlines, these effects persist. This suggests that when making a choice between two competing food options, perceptual salience can play a key role.

Unlike Experiment 1, we observed a sequence effect such that if participants made a healthy decision in the previous trial, the decision in the current trial was more likely to be a healthy one as well. This result has important implications for understanding eating behavior. For example, this may suggest a possible mechanism for habit-forming in healthy eating behavior. That is, it may become easier to make healthy eating choices once a person starts making healthy eating choices. However, further research is necessary to understand what causes these sequence effects better.

Finally, we observed two modest response time effects in which the pattern of data suggested that responses were faster when contextual factors, such as salience or taste preference, favored the healthy choice. It is possible that conflict is reduced when the healthy item is easier to make, either because it has a preferred taste or because it is perceptually salient. However, these are relatively small effects that we did not explicitly predict, and thus, we are cautious in drawing any strong conclusions about response times. Rather, we consider these interesting preliminary results to be a useful starting point for future targeted research.

## General discussion

The results from these experiments provide evidence for the robust effects of salience on food decision-making. Participants were consistently more likely to select the healthier of two food options when it was made more visually salient. This robust pattern was observed across multiple experimental conditions, suggesting that even with additional constraints of cognitive load or time pressure, making healthy food options more salient will lead participants to select healthy food options more frequently.

These findings are important for understanding the mechanisms in how people decide what to eat. There is a well-established role of visual salience biases in driving target selection, not only in general decision-making but also in the context of food decision-making (Armel et al., 2008; Brascamp, Blake, & Kristjánsson, 2011; Milosavljevic et al., 2012; Theeuwes, 1992). However, here we extend these findings to show that even when multiple other relevant dimensions of a food option are manipulated, such as its taste and its healthiness, salience still exerts a robust and consistent effect on choice.

Why would salience impact food decisions? Previous research has established that salience can directly impact attentional selection (e.g., Itti & Koch, 2001; Theeuwes, 1992) and that an attended stimulus is processed in more detail than an unattended stimulus (e.g., Treisman & Gelade, 1980). Furthermore, previous research has found that the taste of a food item can be affected by contextual factors such as hunger (e.g., Burton, Rolls, & Mora, 1976) or lighting (e.g., Scheibehenne, Todd, & Wansink, 2010; for a review, see e.g., Spence, Harrar, & Piqueras-Fiszman, 2012). Thus, the present results suggest that contextual factors can affect not only taste but also the decisions related to food. Whether these changes in decision-making are a result of changes to the anticipated perceived taste of the food remains an open question that we cannot answer in the current experiment. However, the small salience manipulations we conducted in this study changed food choices to a similar degree as active cognitive reappraisal (Boswell et al., 2018) and inhibitory control training (Lawrence et al., 2015; Meule et al., 2014), both of which require more time and effort to achieve comparable reductions in unhealthy food decisions. The ability to train individuals to reduce undesirable eating behavior through a more cost-effective approach, such as the exogenous manipulation of salience, may prove critical for developing effective behavioral interventions.

The present results add further evidence to the notion that salience can affect preference decision-making (e.g., Krajbich, Lu, Camerer, & Rangel, 2012). An open question remains with respect to the generalizability of these salience effects. For example, while some have argued that salient distractors automatically capture attention (e.g., Theeuwes, 1992), there is abundant evidence to suggest that capture is dependent on additional factors, such as task goals or recent experience (e.g., Bacon & Egeth, 1994; Folk et al., 1992; Leber & Egeth, 2006). In other words, top-down control may be able to reduce the extent to which perceptually salient objects capture attention. Thus, the effects of salience on preference decision-making may have similar constraints. For example, previous evidence suggests that salient distractors have reduced effects when they can be anticipated (e.g., Moher, Abrams, Egeth, Yantis, & Stuphorn, 2011; Müller, Geyer, Zehetleitner, & Krummenacher, 2009). Thus, it is possible that salience effects on food decisions might be reduced if salient items appear often. Furthermore, although previous studies have found that salient food objects can capture eye movements in similar paradigms (e.g., Nijs, Muris, Euser, & Franken, 2010), we did not directly measure eye movements or the focus of attention in the current study. Thus, alternative explanations may be possible. For example, it is plausible that increases in the salience of a food item also increases the speed of processing of that food item.^6^ Note that this and other possible explanations do not necessarily exclude that attention capture plays a role, but rather suggest the possibility of one or multiple mechanisms by which perceptual salience changes food choices. Future experiments based on the current paradigm that directly measure the focus of attention via eye-tracking or other means would provide useful additional information on exactly how the salience manipulation affects food-related decision-making.

The second takeaway from these findings is that decisions about which food to choose were predicted by the decision that was made on the preceding trial. When participants made a healthy food decision, their subsequent food decision was more likely to be a healthy one as opposed to an unhealthy one. Note that because this was not a randomly assigned variable, this pattern is correlational. We do not know the causal origin of these effects. Furthermore, while there was a robust main effect in Experiment 2, this pattern was not statistically significant in Experiment 1, though this was likely a power issue as the effect size was similar across experiments. Thus, this appears to be a much weaker effect relative to the salience effect. Nevertheless, the observation of this pattern contributes to our understanding of how people make decisions about food.

A sequence effect in either direction would have been a plausible result. Participants may have wanted to make an equal number of healthy and unhealthy food decisions, in the same way that people in real-world situations will treat themselves with unhealthy foods after eating healthily. However, instead, we observed a pattern of making consecutive healthy (or unhealthy) food decisions, which suggests that previous food decisions are important for setting a trend for future food decisions. One possible explanation of this effect is that it reflects ongoing fluctuations in cognitive control (e.g., Esterman, Noonan, Rosenberg, & DeGutis, 2012; Leber, 2010). In other words, there may have been periods during which participants were better able to make healthy choices, but the cause of any such fluctuations is unknown. More research is needed to target this effect of repeated decision-making, but the result is promising for uncovering mechanisms of decision-making in eating behavior and elucidating the role that habit formation may play.

One limitation of the present study is that we did not include complex food decisions. Given that people will frequently encounter more complex dishes and options than the single-ingredient stimuli (one orange) or single-product stimuli (one Kit Kat bar) we used in our choice tests, a reasonable follow-up study could test the consistency of the salience, taste, and sequence effects we found in more complex food environments that better mimic environments where real food decisions are made. This could include restaurants or grocery stores, where other physical stimuli such as background music (North, Hargreaves, & McKendrick, 1999) and product packaging and labels (Piqueras-Fiszman, Velasco, Salgado-Montejo, & Spence, 2013) may influence decision-making.

There are also many other ways to test the limits of these effects. Is salience relevant only when the difference in taste preference is small? Will it be less important when people are deciding between foods that have large taste rating differences? Because we created food decisions between foods that differed in taste value by only one point, it may be possible that additional sensory information plays a weaker role when decisions are defined by reduced taste conflict. Therefore, knowing the point at which salience is overridden by taste will be useful in capitalizing on the salience effect in food branding, promotions, and packaging. For example, if a participant hates chocolate cake (rated 1) and loves bananas (rated 5), it seems plausible that salience would have little to no effect on their decision. Nevertheless, because grocery stores and restaurants typically offer a plethora of choices, many real-world decisions likely involve options that differ only by small degrees in taste. Those small degrees of taste may also play a particularly important role when a person is deciding between a more or less healthy option. Furthermore, there may be inter-individual differences about the perceived healthiness of individual food items. Although our categories fit general definitions of what is healthy or unhealthy (e.g., candy bars and cakes vs. fruits and vegetables), we did not ask our participants how they rated these foods in terms of healthiness. This may be an important consideration in future research.

Despite these limitations, the present results do have important potential implications for interventions designed to promote and sustain healthy eating. Salience is often easy to manipulate. One could imagine that grocery stores and restaurants, if motivated to help people make healthy food choices, could change the way that foods are presented. Similarly, people may be able to become more active in their own interventions, whether through how they construct their food environments or through augmented reality applications on their phones. Finally, the combination of salience and sequence effects may be important. If salience is leveraged to create a bias towards healthy food decisions for a short time, then perhaps after the salience manipulation is terminated, healthy food decisions would continue to be made. This could also be tested in future experiments, as other research has shown that economic decisions are better predicted using both visual salience and value computation (Towal, Mormann, & Koch, 2013).

Obesity and other eating-related public health concerns are prevalent worldwide. In the present experiment, we have established that perceptual salience can be used to influence decisions towards or away from healthy choices when people are faced with food decisions between choices that vary in tastiness and healthiness. Furthermore, we have shown that when a single decision is made for an unhealthy or healthy choice, this can predict how subsequent decisions will be made. These results contribute to our understanding of food-related decision-making behavior. Though more research is needed, these findings also hold promise for the future development of interventions aimed at promoting healthy eating behavior.

## Data Availability

The datasets generated and analyzed during the current study are available from the OSF repository, https://osf.io/8q259/.

## Footnotes

1 We did not collect demographic information for Experiment 1.1, but general demographic characteristics of Mechanical Turk users can be found in Paolacci and Chandler (2014).

2 Because of a coding error, these participants were not tracked; this was resolved for Experiment 2.

3 Since data were collected online, we had no way to measure absolute brightness experienced by participants, and presumably, display settings differed across participants.

4 We concluded the experiment with two surveys: the Three-Factor Eating Questionnaire-R18 (TFEQ-R18) (de Lauzon et al., 2004) and the Consumer Oriented Nutrition Knowledge (CONK) questionnaire (Dickson, Spillmann, Siegrist, & Keller, 2011). These were intended for exploratory analyses of individual differences. However, no meaningful patterns emerged consistently across the experiments, so we have not included a discussion of these measures in the present manuscript.

5 We ran pilot versions of the cognitive load and reaction-time deadline conditions, which had the same general pattern of results. These are also available from the OSF website.

6 We thank an anonymous reviewer for pointing this out.
